# Label-Free Separation of Circulating Tumor Cells and Clusters by Alternating Frequency Acoustic Field in a Microfluidic Chip

**DOI:** 10.3390/ijms24043338

**Published:** 2023-02-07

**Authors:** Yan Zhang, Ziang Zhang, Dongbang Zheng, Tuchen Huang, Qibin Fu, Yang Liu

**Affiliations:** Sino-French Institute of Nuclear Engineering and Technology, Sun Yat-sen University, Zhuhai 519082, China

**Keywords:** acoustofluidic chip, alternating frequency standing wave, circulating tumor cells, cancer diagnosis

## Abstract

Circulating tumor cells (CTCs) play an important role in the prognosis and efficacy evaluation of metastatic tumors. Since CTCs are present in very low concentrations in the blood and the phenotype is dynamically changing, it is a great challenge to achieve efficient separation while maintaining their viability. In this work, we designed an acoustofluidic microdevice for CTCs separation based on the differences in cell physical properties of size and compressibility. Efficient separation can be achieved with only one piece of piezoceramic working on alternating frequency mode. The separation principle was simulated by numerical calculation. Cancer cells from different tumor types were separated from peripheral blood mononuclear cells (PBMCs), with capture efficiency higher than 94% and a contamination rate of about 1% was obtained. Furthermore, this method was validated to have no negative effect on the viability of the separated cells. Finally, blood samples from patients with different cancer types and stages were tested, with measured concentrations of 36–166 CTCs per milliliter. Effective separation was achieved even when the size of CTCs is similar to that of PBMCs, which has the prospect of clinical application in cancer diagnosis and efficacy evaluation.

## 1. Introduction

Ninety percent of cancer patients’ deaths are caused by cancer metastasis [1]. Circulating tumor cells (CTCs) refer to individual tumor cells in peripheral blood, which play an important role in cancer metastasis [2]. Other studies have shown that CTC clusters, which contain more than two CTCs [3], have 23–50 times stronger capability of metastasis than that of a single CTC [4]. More than 50% of cancer metastases (97% in breast cancer [5]) are caused by CTC clusters [4,6]. Given their importance in cancer metastasis, CTCs and their clusters are often used as markers for the early detection of metastatic tumors and evaluation of efficacy [7,8,9,10]. Studies have found that in an early breast cancer cohort, CTCs can predict the benefit of radiotherapy on patients’ overall survival [7]. Therefore, CTCs are an essential target for liquid biopsy.

Living CTCs can provide a wealth of biochemical information (epithelial and mesenchymal phenotypes, genetic information, etc.) and subsequent testing of drug efficacy, but the concentration of CTCs in the blood is very low (about a few to several hundred cells per milliliter) [11]. This therefore requires separation methods with high efficiency, high purity, and no cell damage. At present, the methods for separating CTCs are mainly classified into two categories: label-based methods and label-free methods. Label-based separation methods depend on CTCs-specific surface markers such as antibodies and aptamers [12]. For example, the FDA-approved CELLSEARCH CTCs Kit was designed to separate CTCs of epithelial origin (CD45^−^, epithelial cell adhesion molecule EpCAM^+^, cytokeratin 8, 18+ and/or 19+) [10,13]. However, some studies have found that not all CTCs expressed the epithelial markers [14,15]. Moreover, the epithelial and mesenchymal composition of CTCs would change dynamically during disease development and treatment [16]. The label-free separation methods do not have this limitation as they rely on the more stable physical properties of the cell, such as size, density, electrical properties, etc. According to the differences in physical properties between CTCs and blood cells, label-free methods include filtration [17], centrifugation [18], inertial focusing [19], and dielectrophoresis [20], etc. However, these methods also have disadvantages, such as being prone to clogging, resulting in cell damage and relatively low efficiency and purity. Therefore, it is of great significance to develop a non-destructive CTCs separation technique with high efficiency and purity.

In recent years, the acoustofluidic method has shown advantages for separating CTCs [21,22,23]. Since the acoustic radiation force is a non-contact force, it would not change cell properties and viability, which was confirmed by our previous study [24]. The basic principle is that cells with different sizes, densities, and compressibilities are subjected to different forces under an acoustic field, thus entering different streamlines to achieve separation. Separation was achieved with a single frequency acoustic field, but requiring special microchannel structure for pre-alignment [25]. Both pre-alignment and separation can be achieved using two piezoelectric ceramics at different positions of the microchannel [26], at the expense of additional components and system complexity In this paper, we proposed a novel method for CTCs separation by applying an alternating frequency acoustic field to the microfluidic chip with only one piezoelectric ceramic. First, device fabrication and experiment setup were introduced. The separation principle was simulated by numerical calculation. Then, cancer cells from three different tumor types (breast cancer cell line MCF7, lung adenocarcinoma cell line A549, and colon cancer cell line HCT116) were separated from peripheral blood mononuclear cells (PBMCs), and the capture efficiency and contamination rate were quantitatively analyzed. The different factors affecting the separation effect were studied. In addition, the effect of the separation process on cell viability was also investigated. Finally, blood samples from tumor patients with different cancer types and stages were tested and the separated CTCs were identified by immunofluorescence. The proposed method demonstrates high efficiency and non-destruction for CTCs separation, which will play an important role in cancer diagnosis and therapeutic effect evaluation.

## 2. Results

### 2.1. Cell Size and Compressibility

**Figure 1:**
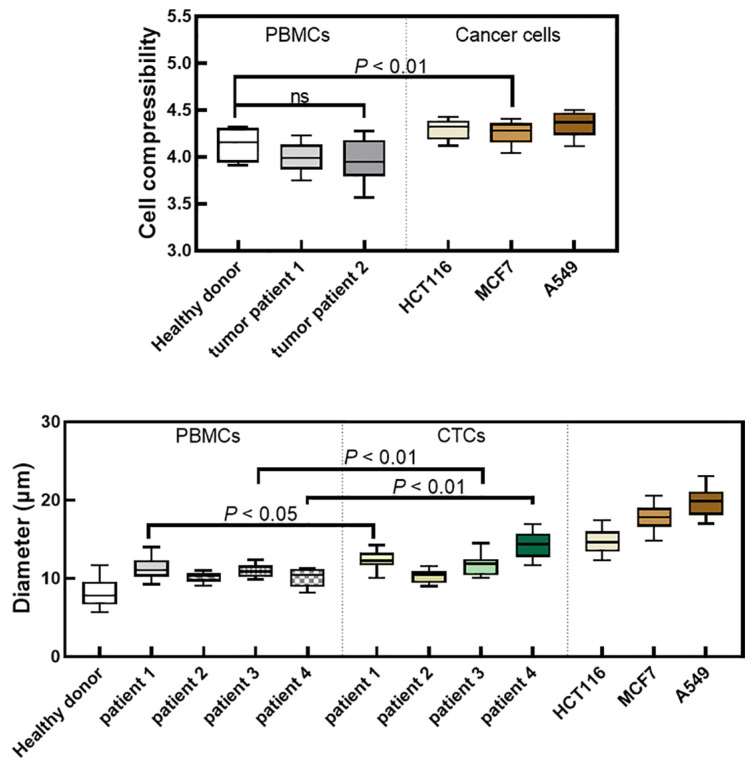
Cell compressibility and diameter of PBMCs from healthy donor, PBMCs from tumor patients, CTCs and cancer cell lines. The unit of cell compressibility was 10^−10^ Pa^−1^. ns indicates not statistically significant.

The measured compressibility and diameter of different cells are shown in Figure 1. The diameter of the cells from cancer cell lines was 14.8–19.6 μm, which was significantly larger than that of the PBMCs from a healthy volunteer. Similarly, CTCs were also larger than PBMCs from patients themselves, although for some patients the differences were less pronounced (Figure 1). Additionally, there was an obvious difference in cell compressibility between cancer cells and PBMCs (Figure 1).

### 2.2. Separation of Cancer Cells from PBMCs

**Figure 2:**
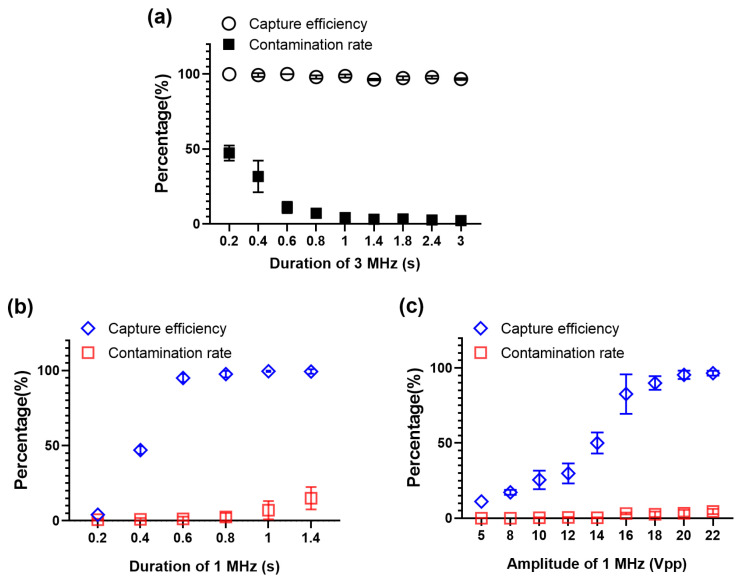
The capture efficiency and PBMCs contamination rate at different duration or amplitude of the 3 MHz or 1 MHz sine wave signal. (**a**) Percentages of MCF7 cells and PBMCs moved to the center of the microchannel at different duration of 3 MHz sine signal. (**b**,**c**) Percentages of MCF7 cells and PBMCs moved to the center of the microchannel at different duration (**b**) or amplitude (**c**) of the 1 MHz sine wave signal. In all figures, values are expressed by means and error bars.

The successful separation of cancer cells from PBMCs is the basis for the separation of CTCs from the blood. Three different cell lines (A549, MCF7, and HCT116) were tested with PBMCs from a healthy volunteer, respectively. The flow rate of the cell channel was fixed at 50 μL/h while the flow rate of the sheath flow was fixed at 100 μL/h. First, the acoustic energy density and duration of the acoustic field for the 3 MHz mode were determined by observing that the cells could be quickly pulled to the standing wave nodes after switching from 1 MHz to 3 MHz. Figure 2a shows the measured capture efficiency and contamination rate for the MCF7 cell line as a function of the duration of the 3 MHz sine signal with a fixed amplitude of 110 Vpp. The duration and amplitude of the 1 MHz sine signal were set to 0.8 s and 9 Vpp, respectively. As the duration increased, the capture efficiency changed little, while the contamination rate decreased significantly and reached a minimum value when the duration was greater than 1 s (Figure 2a).

Then the duration and amplitude of the 1 MHz sine signal was adjusted independently, while the duration and amplitude of the 3 MHz sine signal were set to 1.4 s and 110 Vpp. Figure 2b,c shows the result for the MCF7 cells. As the duration or amplitude of the 1 MHz sine wave signal increased, more cancer cells and PBMCs moved to the midline of the channel, increasing both the capture efficiency and contamination rate. However, the increase in contamination rate was much smaller than the increase in capture efficiency, because the acoustic radiation force for MCF7 cells was stronger than that for PBMCs. Therefore, there is a window in which high separation efficiency can be achieved with low contamination rate.

Table 1 summarizes the representative results for all the three cell lines. With appropriate parameters, the separation efficiency can be as high as 95%, while the contamination rate is about 1%. Although the three cell lines differed in size and compressibility (Figure 1), a window for high efficiency and low contamination rate could be obtained with different parameters (Figure S1).

**Figure 3:**
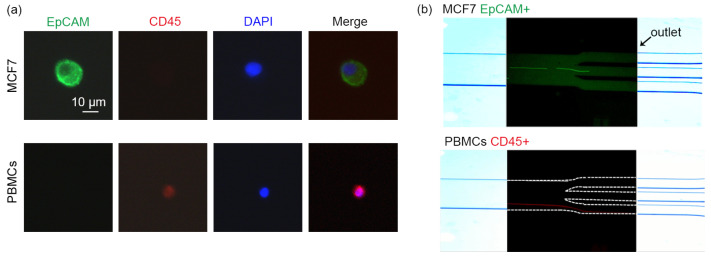
Characterization of cancer cells and PBMCs by immunofluorescence. (**a**) Expression of epithelial biomarkers EpCAM (green) and pan-leukocyte marker CD45 (red) in MCF7 cells and PBMCs. The cell nucleus was stained with DAPI dye (blue). Scale bar = 10 μm. (**b**) Images of fluorescently labeled cancer cells (green) as well as PBMCs (red) flowing through different outlets of the microfluidic chip.

Although it is easy to distinguish cancer cells from PBMCs by size under the microscope, the separation process was further observed with staining. Positivity for epithelial biomarkers (e.g., EpCAM, CK8, CK18, and CK19) and negativity for CD45 are commonly used as biomarkers of CTCs, whereas leukocytes express CD45 but do not express E-type markers [27,28]. As shown in Figure 3a, MCF7 cells were DAPI^+^EpCAM^+^CD45^−^ while PBMCs were DAPI^+^CD45^+^EpCAM^−^. With the stained cell suspension pumped into the microfluidic channel, the cancer cells labeled with green fluorescence were converged to the midline and flowed out through the collection outlet, while the PBMCs labeled with red fluorescence were restricted to the region around the node at the bottom (y = W/6) and collected by the waste outlet (Figure 3b).

### 2.3. Effects of Flow Rate on Capture Efficiency and Contamination Rate

**Figure 4:**
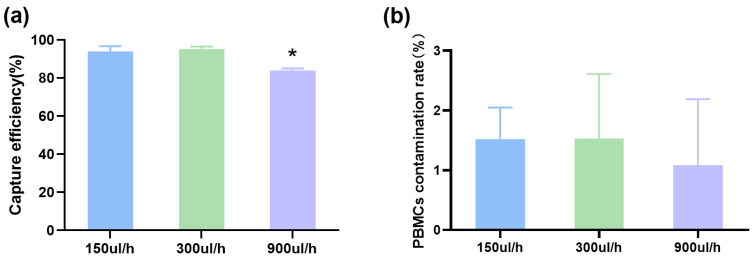
Effects of flow rate on the capture efficiency and contamination rate. The changes of the capture efficiency (**a**) and PBMCs contamination rate (**b**) at different flow rates were compared. * *p* < 0.05.

To evaluate the effect of flow rate on separation process, the mixture of A549 cells and PBMCs were pumped to flow through the acoustofluidic chip at different flow rates of 150 μL/h, 300 μL/h, and 900 μL/h. As shown in Figure 4a, the capture efficiency did not change at flow rates of 150 μL/h (capture efficiency was 94.6 ± 2.7%) and 300 μL/h (capture efficiency was 95.8 ± 1.5%). When the flow rate increased to 900 μL/h, the cell capture efficiency decreased to 84.0 ± 1.0%. However, the PBMCs contamination rate did not change significantly with the increase in the flow rate, maintaining at about 1% (Figure 4b). As the flow rate increased, the time for the cell to flow through the microchannel becomes shorter, so the durations of the acoustic field should be reduced correspondingly.

### 2.4. Evaluation of Cell Viability after Acoustic Separation

**Figure 5:**
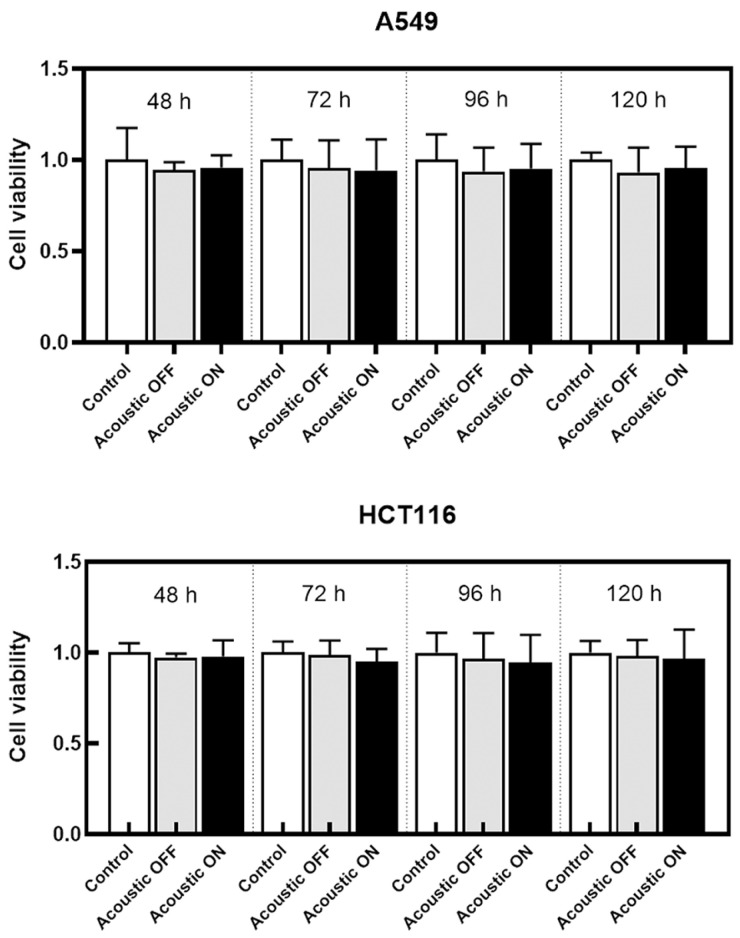
Cell viability at different times after separation. The cell viability of the acoustic-OFF group (gray) and the acoustic-ON group (black) was normalized with the control group (white).

In clinical application, the separated cancer cells should be cultured for further studies such as drug experiments. Therefore, it is necessary to evaluate the effect of the separation process on cell viability. We performed cell proliferation assays using A549 and HCT116 cell lines. The flow rate was set to 400 μL/h. Two groups of cells were tested on the microfluidic chip with and without the acoustic field. Cells were cultured and counted at 48, 72, 96, and 120 h after the test. As shown in Figure 5, for both the A549 cells and the HCT116 cells, there was no significant difference in cell viability between the control group, the acoustic-off group, and the acoustic-on group at each measurement time. The results indicated that the proposed method had no effect on cell viability and proliferation.

### 2.5. Separation of CTCs from Tumor Patients

**Figure 6:**
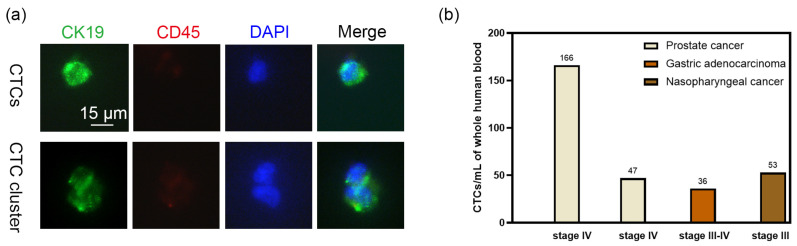
Identification of CTCs and cluster from tumor patients. (**a**) Representative immunofluorescence images of isolated CTCs and cluster. CTCs and CTC cluster were stained with cytokeratin 19 (green), CD45 (red) and DAPI (blue). Scale Bar = 15 μm. (**b**) The number of captured CTCs per milliliter of whole blood for each tumor patient.

To verify the possibility of clinical application of our method, we performed double-blind CTCs separation from four patients with different types and stages (stage III~IV) of cancer. The flow rate was increased to 900 μL/h because of the large volume of samples tested. After separation, the cells were identified by staining with epithelial cell marker (CK19), pan-leukocyte marker (CD45), and nuclear marker (DAPI). CTCs were identified with CK19^+^CD45^−^DAPI^+^ (Figure 6a and Figure S2). The number of CTCs measured ranged from 36 to 166 CTCs per milliliter for different patients, with a mean value of 75.5 CTCs per milliliter (Figure 6b). In addition, a CTC cluster consisting of two CTCs was observed (Figure 6a), which is consistent with other reports [3].

## 3. Discussion

The American Joint Committee on Cancer (version 2010-v7 and version 2018-v8) has added cM0(i+) staging based on CTCs, which indicates the important role of CTCs in tumor metastasis and staging. In patients with early-stage breast cancer, ≥1 CTCs per 7.5 mL of peripheral blood indicates poor prognosis, and ≥5 CTCs indicate metastatic breast cancer. A large number of clinical studies have shown that CTCs enumeration was valuable in response evaluation and prognosis evaluation of a variety of tumors [7,29,30]. Moreover, the important role of CTCs cluster in tumor metastasis has been gradually discovered [31]. Although the concentration of CTCs is relatively low, making accurate separation of CTCs a great challenge, and its advantage over other liquid biopsy objects (such as ctDNA) is that CTCs are viable intact cells, which can accurately provide genomic information of cancer patients [32]. Recently, studies have isolated CTCs from pancreatic cancer patients and identified new single-cell copy number variations [33]. In addition, patient-derived CTCs cell lines can also be cultivated [34]. Given the clinical importance of CTCs and their potential as research tools, it is important that our method can be used for efficient and nondestructive separation of CTCs.

Our approach is to separate cells based on their size and compressibility. The cell compressibility reflects the bulk modulus, which is an important parameter to describe the mechanical properties of cells [35,36]. Our previous studies found that cell compressibility changed after cells underwent epithelial to mesenchymal transition, and cell compressibility was related to the malignancy of cancer cells [35]. An important feature of our method is the alternating frequency standing wave. If there are differences in size and compressibility between cells, they will experience different amounts of acoustic radiation forces. With the action of alternating frequency standing wave, they converged to different streamlines in the microchip, thus achieving separation. Since established cell lines differ significantly in size and compressibility from a healthy donor’s PBMCs (Figure 1), our device can isolate cancer cells with an efficiency as high as 95%, while the contamination rate of PBMCs was only about 1% (Table 1). Even when the flow rate was increased to 900 μL/h, the separation efficiency was around 84% and the PBMCs contamination rate remained unchanged (Figure 4). Compared with other separation methods based on acoustofluidic, the usual flow rate was 120–6000 μL/h, the separation efficiency of cancer cell lines was 71–98%, and the PBMCs contamination rate was 0.2–10% [25,37,38,39,40,41,42]. It is worth noting that the higher flow rates reported in some studies were due to the larger cross-section of the microchannel (such as five times larger than our [39]) and higher intensity of acoustic field, which will be improved in our next optimization.

In the published literature, the successful application of acoustofluidic method to the separation of CTC is rare, and there was a significant difference in size between the separated CTCs and white blood cells (WBCs), which were relatively easy to separate. However, not all patients exhibited significant difference in the size of CTCs and PBMCs (Figure 1). On the one hand, there are individual differences between each person, and even WBCs from healthy donors shows a wide range in size (8–15 μm) [43]. On the other hand, tumors were inherently heterogeneous. Therefore, this presents some challenges for approaches that achieve separation based on difference in cell size only. Nonetheless, a difference in Young’s modulus between CTCs and WBCs has been reported in the literature [44,45,46]. In the existing acoustic separation methods, our device was the first to successfully separate CTCs from PBMCs with similar sizes. Moreover, more CTCs (about 75 CTCs per milliliter) was detected compared to other acoustofluidic methods. This indicated that the sensitivity of our method was relatively high.

The current limitation of our method is mainly the flow rate, which can be adjusted but will affect the separation effect. Compared to other studies based on the acoustofluidic method, the flow rate in our study is at a moderate level. Although a higher flow rate was reported in some studies, the leukocyte contamination rate was also significantly higher than our result. It is also worth mentioning that the high flow rate reported in some studies was due to the larger cross section of the micro-channel and higher intensity of the acoustic field, which will be improved in our next optimization. Furthermore, the CTC cluster may be destroyed if the flow rate is too high, failing to capture it [27]. Therefore, the flow rate, separation efficiency, and PBMCs contamination rate should be comprehensively considered and optimized.

## 4. Materials and Methods

### 4.1. Device Fabrication and Setup

**Figure 7:**
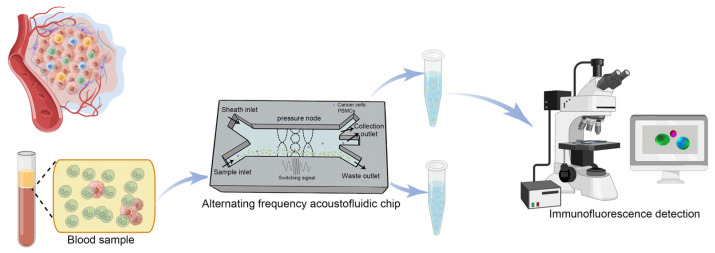
Schematic diagram of the separation process by acoustofluidic chip.

Figure 7 has shown the separation and identification of cancer cells from PBMCs by alternating frequency acoustofluidic microchip. Cancer cells can be derived from a cancer cell line or from the peripheral blood of tumor patients. A microchannel with a rectangular cross-section (737 μm wide and 50 μm deep) was fabricated on a silicon wafer by reactive ion etching. The microfluidic chip channel has two inlets and three outlets (Figure 7). The top of the channel was sealed with a piece of transparent Pyrex and the bottom of the channel was attached with a piezoceramic to provide the acoustic field. The piezoceramic operated in alternating frequency mode with a fundamental frequency of 1 MHz and a harmonic of 3 MHz. Cells and the PBS buffer were pumped into the microfluidic chip channel by syringes from the sample inlet and the sheath inlet, respectively. The signal generator generated two sine wave signals with frequencies of 1 MHz and 3 MHz, which were fed to a self-developed signal switching board. These two signals were switched through a single pole double throw (SPDT) relay, controlled by a square wave signal output by the signal generator. The selected signal was further amplified by a power amplifier before being applied to the piezoceramic. The motions of cells were observed with a microscope and recorded with a CCD camera. The capture efficiency and contamination rate were calculated by analyzing the cells in the Video S1.

### 4.2. Principle of Separation and Simulation

**Figure 8:**
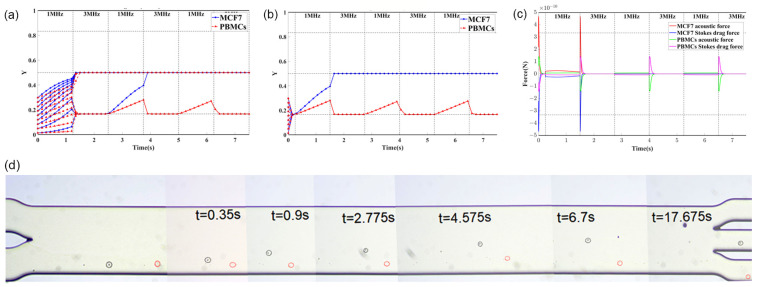
The simulated trajectories of the MCF7 cells and PBMCs entering the acoustic field at different times with y0 < W/3. (**a**) Cells entered the acoustic field at the beginning of the 1 MHz mode. (**b**) Cells entered the acoustic field at the end of the 3 MHz mode. (**c**) Acoustic radiation force and Stokes drag force acting on MCF7 and PBMCs corresponding to the case shown in Figure (**b**). (**d**) Representative images of the separation process of MCF7 cells (black circle) and PBMCs (red circle) in the acoustofluidic chip.

When a standing wave acoustic field was established across the microchannel, the cells would move towards the standing wave node under the action of the acoustic radiation force. As shown in Figure 8a, for the 1 MHz mode, only one node was formed at the midline of the microchannel (i.e., y = W/2). For the 3 MHz mode, three nodes were formed at y = W/6, y = W/2, and y = 5W/6. Therefore, when working in the 1 MHz mode, all the cells moved towards the midline, but the displacements in the y direction were different for cells of different size and compressibility. When in 3 MHz mode, cells moved towards the nodes closest to them, thus achieving separation.

For successful separation, the cells should be aligned before entering the acoustic field. This can be performed by setting a sheath flow rate higher than the sample flow rate so that the cells would be constrained within a small region in y direction. Here, the initial position of the cells in y direction were limited to y0 < W/3 region by setting the ratio of the sample inlet flow to that of the sheath flow to 1:2.

The motion of the cells along y direction of the channel is governed by the acoustic radiation force and the Stokes drag force. With given parameters of the cells and acoustic field, the trajectories of the cells in the microchannel can be simulated by numerical solution using fourth-order Runge–Kutta method, as described in detail in our previous study [35,47]. Figure 8 shows the simulated trajectories of the MCF7 cells and PBMCs entering the acoustic field at different times with y0 < W/3. When cells enter the acoustic field during the 3 MHz mode or at the end of the 1 MHz mode (Figure 8b and Figure S3), the MCF7 cells and PBMCs can be completely separated. Only when cells enter the acoustic field at the beginning of the 1 MHz mode (Figure 8a), a small portion of PBMCs will be concentrated to the midline, which increases the contamination rate. After two switching cycles, the MCF7 cells were pushed to concentrate at the midline of the channel and flowed downstream, while most of the PBMCs were confined to flow around the node (y = W/6) of 3 MHz acoustic field (Figure 8d and Supplementary Video S1). Therefore, they can be collected from the collection outlet and waste outlet, respectively. The acoustic radiation force and the Stokes drag force, corresponding to the case of Figure 8b, are numerically simulated and shown in Figure 8c.

In theory, best separation can be achieved by limiting the initial position to y0 < W/6, but the throughput will be greatly reduced.

Based on this separation principle, the separation effect depends on the acoustic energy densities and durations of the acoustic field for the 1 MHz and 3 MHz mode. Cells must undergo at least two switching cycles in the acoustic field. During 1 MHz mode, the displacement of MCF7 cells in the y direction should be greater than W/6, while that of PBMCs should be less than W/6, thus ensuring that they are pulled to different nodes after switching to 3 MHz mode. During 3 MHz mode, the cells should be pulled back to the nodes as quickly as possible.

### 4.3. Measurement of Cell Compressibility and Size

The difference in size and compressibility of the cells lays the basis for the separation. The size of the cells was obtained by analyzing microscopic images using ImageJ software. The cell compressibility was measured based on a self-developed acoustofluidic microdevice, as described in detail in our previous study [35].

### 4.4. Cell Cultivation

Cell lines were purchased from the American Type Culture Collection. Lung adenocarcinoma cell line A549 was cultured in high glucose (4.5 g/L) Dulbeccos modified Eagle’s medium providing 10% fetal bovine serum (FBS). Breast cancer cell line MCF7 was maintained in the minimum essential medium supplemented with 10% FBS. Roswell Park Memorial Institute (RPMI) 1640 medium containing 10% FBS was used for culturing the colon cancer cell line HCT116. 100 U/mL penicillin and 100 μg/mL streptomycin were added to all complete media. The culture environment was set at 37 °C in 95% air and 5% CO_2_.

### 4.5. Blood Sample Preparation

Human whole blood from healthy donor and tumor patients were collected under the approval of the ethics committee of the Fifth Affiliated Hospital of the Sun Yat-sen University (K175-1(2022)). The polysucrose solution (Sigma-Aldrich, Darmstadt, Germany) was added to the centrifuge tube, then human whole blood was added carefully to the upper layer in a 1:1 ratio. The sample was centrifuged at 400× *g* for 30 min at room temperature. After that, it can be separated into three layers: the lower layer was a mixture of erythrocytes and macromolecular proteins, and the upper layer was plasma. The middle layer was a polysucrose solution and there was a foggy layer at the interface with the upper layer, that is, high-purity PBMCs. Such a foggy interface was transferred to another centrifuge tube with 0.5 mL of the upper layer of plasma and washed with 10 mL PBS containing 1% bovine serum albumin (BSA) at 250× *g* for 10 min. To remove red blood cells remaining in the tube, the cell pellet was resuspended and lysed with 2 mL of red blood cell lysis buffer for 5 min. Then, cells were washed by 10 mL PBS containing 1% BSA. The PBMCs can be resuspended for later use after being washed 2–3 times.

### 4.6. Evaluation of Separation Effect

Capture efficiency (or capture recovery) is defined as the ratio between the number of captured cancer cells (CCs) and the total number of CCs entered in the microfluidic chip.

Capture efficiency = CCs_captured/CCs_in(1)

PBMCs contamination rate is defined as the ratio between the number of captured PBMCs (i.e., the PBMCs mixed with the captured cancer cells) and the total number of PBMCs entered in the microfluidic chip.

PBMCs contamination rate = PBMCs_captured/PBMCs_in(2)

Here, the captured cancer cells or PBMCs were counted as the ones that converged to the midline of the microchannel as observed in the microscope, while the cancer cells and PBMCs were distinguished by size.

The cancer cells were mixed with the PBMCs at a ratio of 1:100. The total number of cancer cells was about 1500. Depending on the experimental conditions, we counted 200 to 500 cells per experiment and repeated the experiment at least three times.

### 4.7. Cell Viability and Proliferation

To evaluate the effect of the microfluidic channel and bulk acoustic wave on cell viability and proliferation, the experiment was divided into three groups: acoustic-on group, acoustic-off group and control group (sham-treated group). Cells were grown to 90% confluent in 60 mm diameter dishes and digested with 0.05% trypsin. Then cells were centrifuged at 1200 rpm and resuspended with the complete medium to prepare a cell suspension. Cells in the control group were directly seeded in 24-well plates after counting. For the acoustic-off group, cells passing through the microfluidic channel with the ultrasonic field turned off were collected and seeded. For the acoustic-on group, cells passing through the microfluidic channel with the ultrasonic field turned on were collected and seeded. Cells were cultured and counted at 48, 72, 96, and 120 h after test.

### 4.8. Identification of Cancer Cells and PBMCs by Immunofluorescence

The suspended cells were fixed with 4% paraformaldehyde for 30 min and washed by centrifugation in PBS. Then cells were resuspended in PBS containing 0.3% Triton X-100 and 5% BSA, and blocked for 1 h. After centrifugation and washing, the cell pellet was resuspended in 3% BSA solution with primary antibodies at room temperature for 1 h. After that, cells were washed twice with PBS. Then the cell pellet was resuspended in 3% BSA solution with the secondary antibodies at room temperature for 1 h. After centrifugation and washing, the resuspended cells were stained with a 1:1 mixture of DAPI solution and 3% BSA solution for 10 min. After washing with the PBS, the sample was standby for observation. The primary antibodies used in this study were CD45 antibody (Cell Signaling Technology, Danvers, MA, USA), EpCAM antibody (Cell Signaling Technology), and cytokeratin 19 antibody (Abcam, Cambridge Science Park, UK). The secondary antibodies were coraLite488 conjugated goat anti-mouse IgG (Proteintech, Chicago, IL, USA), and coraLite594 conjugated goat anti-rabbit IgG (Proteintech). Fluorescence images were taken using a fluorescence microscopy (Olympus, Tokyo, Japan) with a 40× objective lens.

### 4.9. Statistics

During this study, at least three independent replicates were carried out for every experiment. The results are presented as mean ± SD. Significant differences were determined by Student’s *t*-test and Chi-square analysis, defined as *p* < 0.05 or *p* < 0.01 (extremely significant differences).

## 5. Conclusions

In this study, we developed an efficient and non-destructive method for CTCs separation by applying an alternating frequency acoustic field to the microfluidic chip with only one piezoelectric ceramic. Cancer cells from different tumor types were separated from PBMCs, with capture efficiency higher than 94% and contamination rate about 1%. In addition, this method was validated to have no negative effect on the viability of the separated cells. Furthermore, blood samples from patients with different cancer types and stages (III~IV) were tested, with measured concentrations of 36–166 CTCs per milliliter. Compared with other label-free separation methods, this method has the advantages of online separation, high capture efficiency, low contamination rate, and non-destruction. More importantly, effective separation was achieved even when the size of CTCs is similar to that of PBMCs, showing the prospect of clinical application in cancer diagnosis and efficacy evaluation.

## Figures and Tables

**Table 1:** The representative results for three cancer cell lines.

Cell Line	Capture Efficiency	PBMCs Contamination Rate
MCF7	95.0 ± 2.8%	1.3 ± 1.4%
HCT116	94.4 ± 0.5%	1.7 ± 0.3%
A549	94.6 ± 2.7%	1.5 ± 0.5%

## Data Availability

The data presented in this study are available within the article and its Supplementary Data Files.

## Supplementary Materials

The following supporting information can be downloaded at: https://www.mdpi.com/article/10.3390/ijms24043338/s1.
