# Supplementary figures and images for: Label-Free Separation of Circulating Tumor Cells and Clusters by Alternating Frequency Acoustic Field in a Microfluidic Chip
Source: Int J Mol Sci. 2023 Feb 7;24(4):3338. doi: 10.3390/ijms24043338 (PMC9964901; doi:10.3390/ijms24043338)

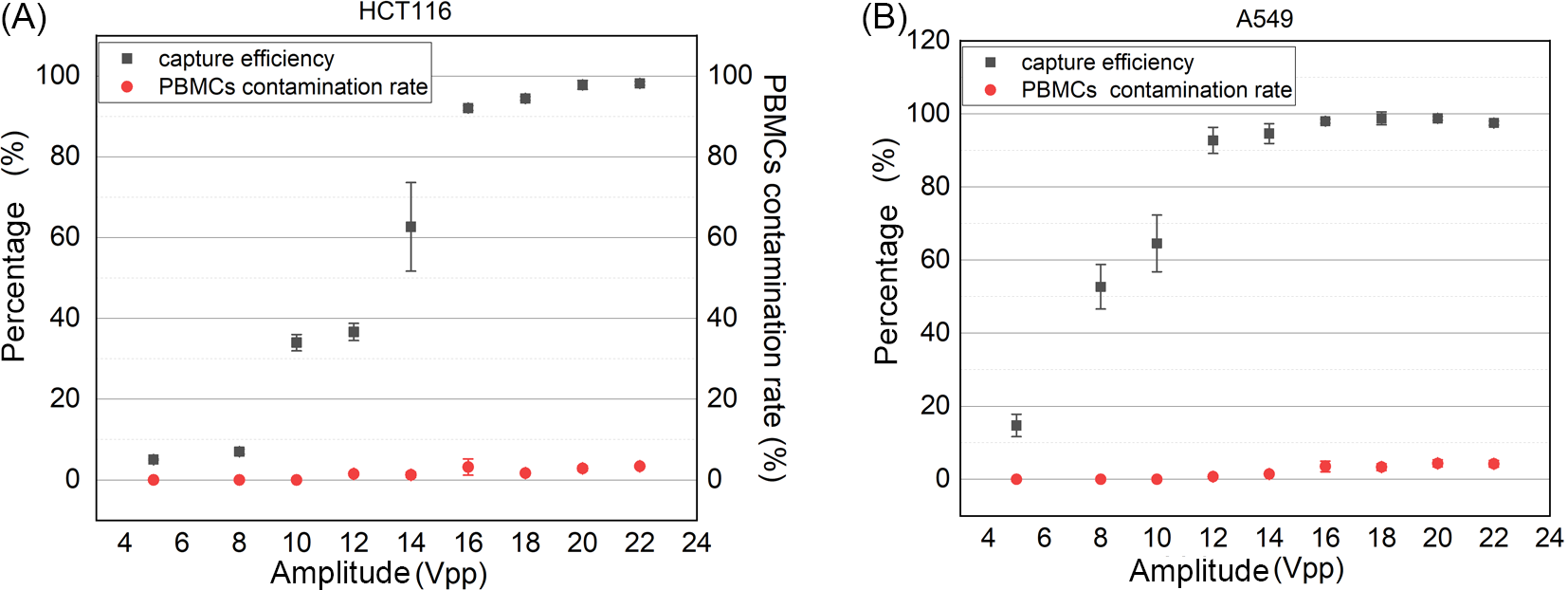

Supplement: Supplementary file 1 [file ijms-24-03338-s001.zip › Figure S1.tif]

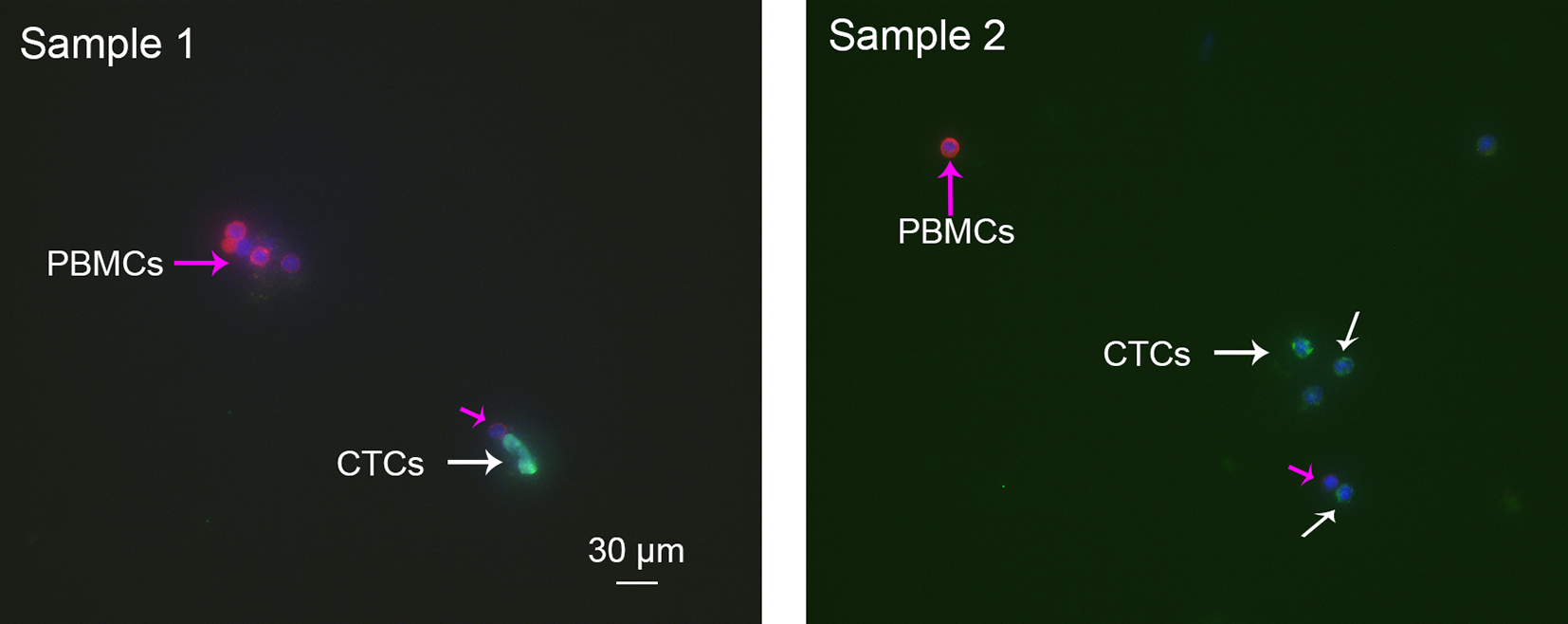

Supplement: Supplementary file 1 [file ijms-24-03338-s001.zip › Figure S2.tif]

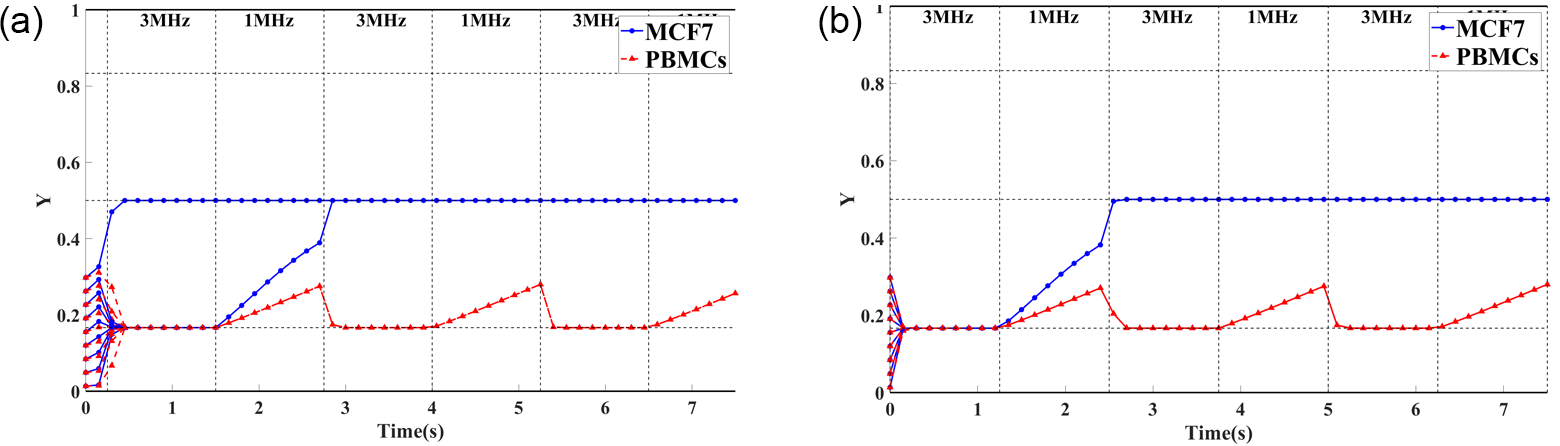

Supplement: Supplementary file 1 [file ijms-24-03338-s001.zip › Figure S3.tif]
